# Femtosecond laser-based nanosurgery reveals the endogenous regeneration of single Z-discs including physiological consequences for cardiomyocytes

**DOI:** 10.1038/s41598-019-40308-z

**Published:** 2019-03-06

**Authors:** Dominik Müller, Dorian Hagenah, Santoshi Biswanath, Michelle Coffee, Andreas Kampmann, Robert Zweigerdt, Alexander Heisterkamp, Stefan M. K. Kalies

**Affiliations:** 1 Institute of Quantum Optics, Leibniz University Hannover, Hannover, Germany; 2 REBIRTH-Cluster of Excellence, Hannover Medical School, Hannover, Germany; 3 Lower Saxony Centre for Biomedical Engineering, Implant Research and Development (NIFE), Hannover, Germany; 4 Leibniz Research Laboratories for Biotechnology and Artificial Organs (LEBAO), Department of Cardiac, Thoracic, Transplantation and Vascular Surgery (HTTG), Hannover Medical, School, Hannover, Germany; 5 Clinic for Cranio-Maxillo-Facial Surgery, Hannover Medical School, Hannover, Germany

## Abstract

A highly organized cytoskeleton architecture is the basis for continuous and controlled contraction in cardiomyocytes (CMs). Abnormalities in cytoskeletal elements, like the Z-disc, are linked to several diseases. It is challenging to reveal the mechanisms of CM failure, endogenous repair, or mechanical homeostasis on the scale of single cytoskeletal elements. Here, we used a femtosecond (fs) laser to ablate single Z-discs in human pluripotent stem cells (hPSC) -derived CMs (hPSC-CM) and neonatal rat CMs. We show, that CM viability was unaffected by the loss of a single Z-disc. Furthermore, more than 40% of neonatal rat and 68% of hPSC-CMs recovered the Z-disc loss within 24 h. Significant differences to control cells, after the Z-disc loss, in terms of cell perimeter, x- and y-expansion and calcium homeostasis were not found. Only 14 days *in vitro* old hPSC-CMs reacted with a significant decrease in cell area, x- and y-expansion 24 h past nanosurgery. This demonstrates that CMs can compensate the loss of a single Z-disc and recover a regular sarcomeric pattern during spontaneous contraction. It also highlights the significant potential of fs laser-based nanosurgery to physically micro manipulate CMs to investigate cytoskeletal functions and organization of single elements.

## Introduction

The cellular cytoskeleton represents a complex ubiquitous element across all cell types. It defines the shape, determines mechanical properties, and affects signaling pathways^1,2^. In particular, cardiomyocytes (CMs) have a highly organized cytoskeletal architecture, which is suited to facilitate the maximal systolic output^2^. A part of the CM cytoskeleton is responsible for force generation and build-up of sarcomeres, which are the smallest contractile units of myocytes. Actin and myosin filaments are arranged in each sarcomere. In response to increased calcium levels, the well-controlled cross-bridge cycle between actin and myosin filaments leads to force generation and muscle shortening^3,4^. Single sarcomeres in this sarcomeric cytoskeleton are connected at the lateral ends via Z-discs. The Z-disc is a hetero molecular structure with a width of 100–140 nm, composed of mostly α-actinin layers^5^. Beside α-actinin, more than 124 human genes or gene products are associated with the Z-disc which are involved in contractibility, gene regulation^6^, gene transcription^7,8^, cell-fate determination^9^ and survival in CMs^10,11^.

Patients with hypertrophic^12–15^, dilated^3,16–19^, arrhythmogenic right ventricular^20^, or restrictive cardiomyopathies^21^ show altered or disrupted sarcomeric elements or Z-disc structures^2,5^. Several transgenic animal models were generated to analyze mutations in cytoskeletal elements^6,18^. Genetic modifications (e.g. knock-out or overexpression) as well as chemical treatments^22,23^ were applied to reveal the physiological functions of cytoskeleton elements *in vitro*. However, these methods equally affect all related cellular elements and fail to elucidate the function of a single structure in the whole ensemble of cytoskeletal elements. Furthermore, it is impossible to determine different functions of distal or central cytoskeletal elements, for instance, in case of the Z-disc. A tool to better identify the fundamental function of single Z-discs in a disease context versus appropriate controls is therefore attractive to investigate respective disease mechanisms in a dish. This may also support studies on pharmacological treatments and elucidate their potential effect(s) on cell regeneration versus disruption thereof.

To have a complete understanding of the function and role of single or isolated cytoskeletal structures, in particular in the sarcomeric cytoskeleton, it is necessary to either precisely prevent their function or to remove the structure. Laser based microsurgery can be a potent tool to achieve manipulation of cellular structures^24^. However, the cellular response to laser irradiation depends on a complex interplay of laser wavelength, intensity, applied energy density, and exposure time. Even low dose laser irradiation can induce cellular effects by triggering the accumulation of superoxide, while high laser doses lead to elevated levels of reactive oxygen species production^25^. To limit the exposure time, pulsed laser systems can be used. Femtosecond (fs) laser-based system are the most promising tool for cellular nanosurgery^26–30^. Through the nonlinear interactions with tissue, fs laser pulses enable a localized, confined, and contact-free ablation tool to remove cellular elements^26–28,30^. The dissection is based on the formation of a so called “low-density plasma” of free electrons through multiphoton ionization, which can lead to bond-breaking in biomolecules^28,30^. The multiphoton ionization process is limited to the focus because of the necessary photon density. Additionally, reactive oxygen species might be formed, as observed in the case of MHz repetition rate laser systems by irradiation of the nucleus. In certain cell regions, this might change the cellular response^31^. However, no significant thermal and mechanical energy is transferred to the surrounding environment, which guarantees precise operation^27,30^. Due to complexity, throughput, and costs of operating a fs laser system, this approach has rarely been used in studies of cellular repair or mechanobiology^27,29,32,33^. Examples using the technique include the ablation of mitochondria^27^ or nanosurgery in actin stress fibers^29,33^.

**Figure 1:**
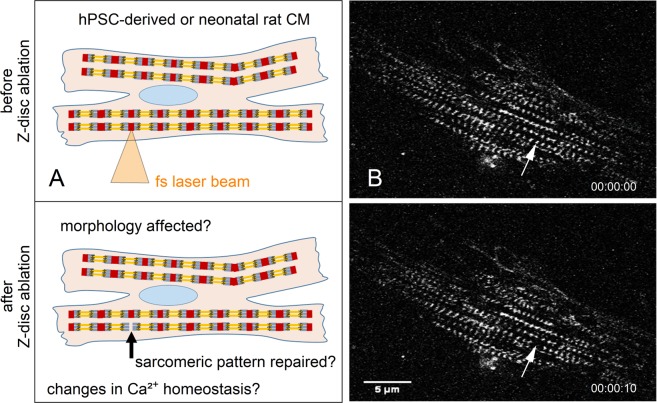
Femtosecond laser nanosurgery was used to ablate a single Z-disc in cardiomyocytes. The illustration (**A**) depicts the ablation process, which is visible in (**B**) multiphoton microscopy images of a turboRFP linked α-actinin expressing neonatal rat cardiomyocyte. The arrow indicates the position of the selected Z-disc before (top right) and 10 s after ablation (bottom left). Scale bar 5 µm.

For the first time, we utilized the advantages of fs laser-manipulation to investigate cell homeostasis, alterations, and repair after selective microdamage in the sarcomeric cytoskeleton. In particular, we aimed to understand the role of a single Z-disc in the complex cytoskeletal network in spontaneous contracting CMs. A single Z-disc per CM was ablated (Fig. 1), which gave the opportunity to assess the immediate morphological changes and reveal a possibly altered calcium homeostasis within the whole cell. In addition, we analyzed the endogenous repair potential of treated CMs over time. CMs differentiated from human pluripotent stem cells (hPSC) and neonatal rat CMs were compared. Stem cell-derived cardiomyocytes represent an important model in the field of tissue engineering of cardio vascular constructs and are a potential cell source for transplantation therapies, drug discovery, or disease modelling^34^. Neonatal rat CMs are an established native comparison for this study^35^. As maturation of cells in culture could change the cytoskeletal mechanics, all analyses were performed at different points in time (days *in vitro*, DIV) after cell isolation or differentiation.

## Results

### Cardiomyocyte viability is unaffected by loss of a single Z-disc

**Figure 2:**
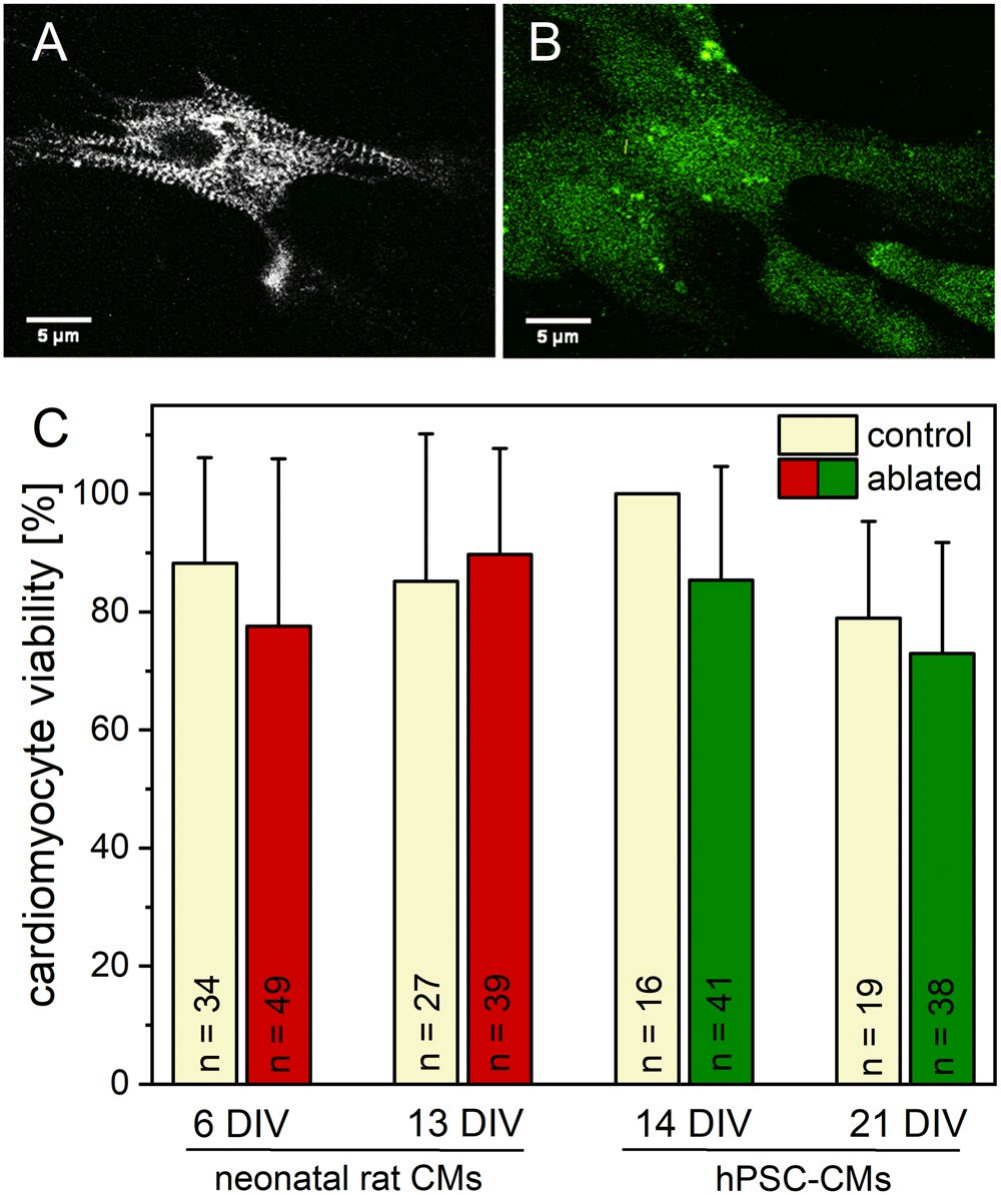
Viability of CMs after single Z-disc ablation. 24 h after laser treatment, the metabolic activity was visualized using Calcein-AM staining. The multiphoton image of a turboRFP linked α-actinin expressing hPSC-CM before (**A**) and after Calcein-AM staining (**B**). Previous randomly selected untreated CMs served as control group. No major decrease in cell viability was observed for all analyzed conditions (**C**). Bars represent mean viability + standard deviation.

In an initial series of experiments, we determined the viability of CMs after ablation of single Z-disc in the sarcomeric cytoskeleton. As each Z-disc serves as a connection between adjacent sarcomeres, and cannot be regarded as isolated, we expected an influence on cell metabolism and viability. We compared the manipulated CMs with single Z-disc ablation to untreated CMs. The cell metabolism was visualized 24 h after Z-disc ablation with Calcein-AM. The metabolic activities in treated and untreated cells were comparable, with ≥73% of viable hPSC-CMs and ≥78% of viable neonatal rat CMs (Fig. 2). Some Calcein-positive, fluorescent cells showed a spherical morphology with stationary protrusions of the cell membrane (Supplementary Fig. S1), which indicates cell death^36^. These CMs were counted as dead cells. Adjacent cells were unaffected by laser treatment.

### The sarcomeric pattern is restored in half of the cells

**Figure 3:**
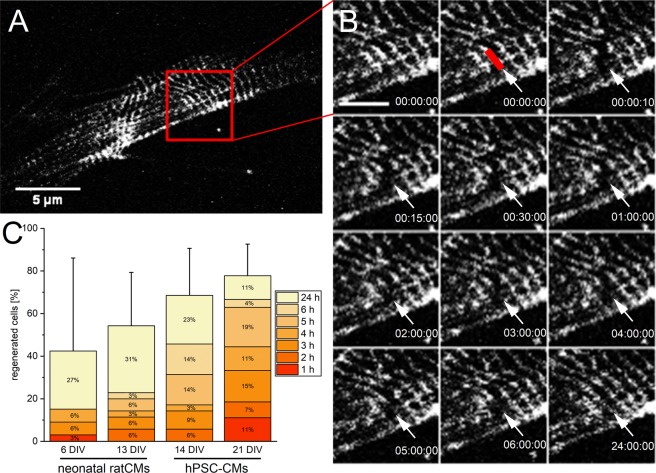
Endogenous repair of disrupted Z-disc pattern. A representative image of the Z-disc pattern of a neonatal rat CM before fs laser nanosurgery (**A**, scale bar 5 µm). The ablation of a single Z-disc and a time series until the regular pattern was recovered after 24 h (**B**). The red line indicates the ablated Z-disc. Scale bar 2 µm. The stacked bar chart (**C**) depicts the percentage of CMs + standard deviation, which recovered a regular pattern within 24 h after single Z-disc ablation. The distribution of how many cells recovered the Z-disc pattern at a decent time point is presented within the stacked bar chart. 6 DIV n = 33, 13 DIV n = 35, 14 DIV n = 35, 21 DIV n = 27.

The ability for sufficient endogenous repair after single Z-disc ablation was analyzed. As the viability of cells was almost unaffected, we expected a successful recovery of the sarcomeric cytoskeleton including the ablated Z-disc. Therefore, the manipulated sarcomeric pattern was compared to the original pattern over a time period of 24 h after laser treatment (Fig. 3). More than 68% of viable 14 DIV old hPSC-CMs showed a recovered Z-disc pattern. In 21 DIV old CMs, the recovery rate was 78%. A lower repair potential was found in 6 DIV (42%) and 13 DIV (54%) old neonatal rat CMs. In addition, already 1 h to 2 h post ablation, a few CMs recovered their Z-disc pattern. However, the majority of treated CMs recovered a regular Z-disc pattern after 5 h (hPSC-CMs) or 24 h (neonatal rat CMs).

To exclude photobleaching as a nanosurgery artifact, we performed fluorescence recovery after photobleaching (FRAP) measurements of intact sarcomeres. We observed recovery of α-actinin fluorescence to a saturated value with a half-recovery time of 200 s (Supplementary Fig. S2, exponential fit).

### Changes in cell morphology can accompany the loss and repair of a single Z-disc

Each Z-disc in CMs is connected to the whole cytoskeleton framework over adjacent sarcomeres and costamers^2^. Therefore, we hypothesized that the loss and repair of a single Z-disc may be accompanied by changes of the cell morphology and cell cytoskeleton. To investigate these changes, we determined the cell area, perimeter, and the length of cell’s major and minor axis before and after single Z-disc ablation.

**Figure 4:**
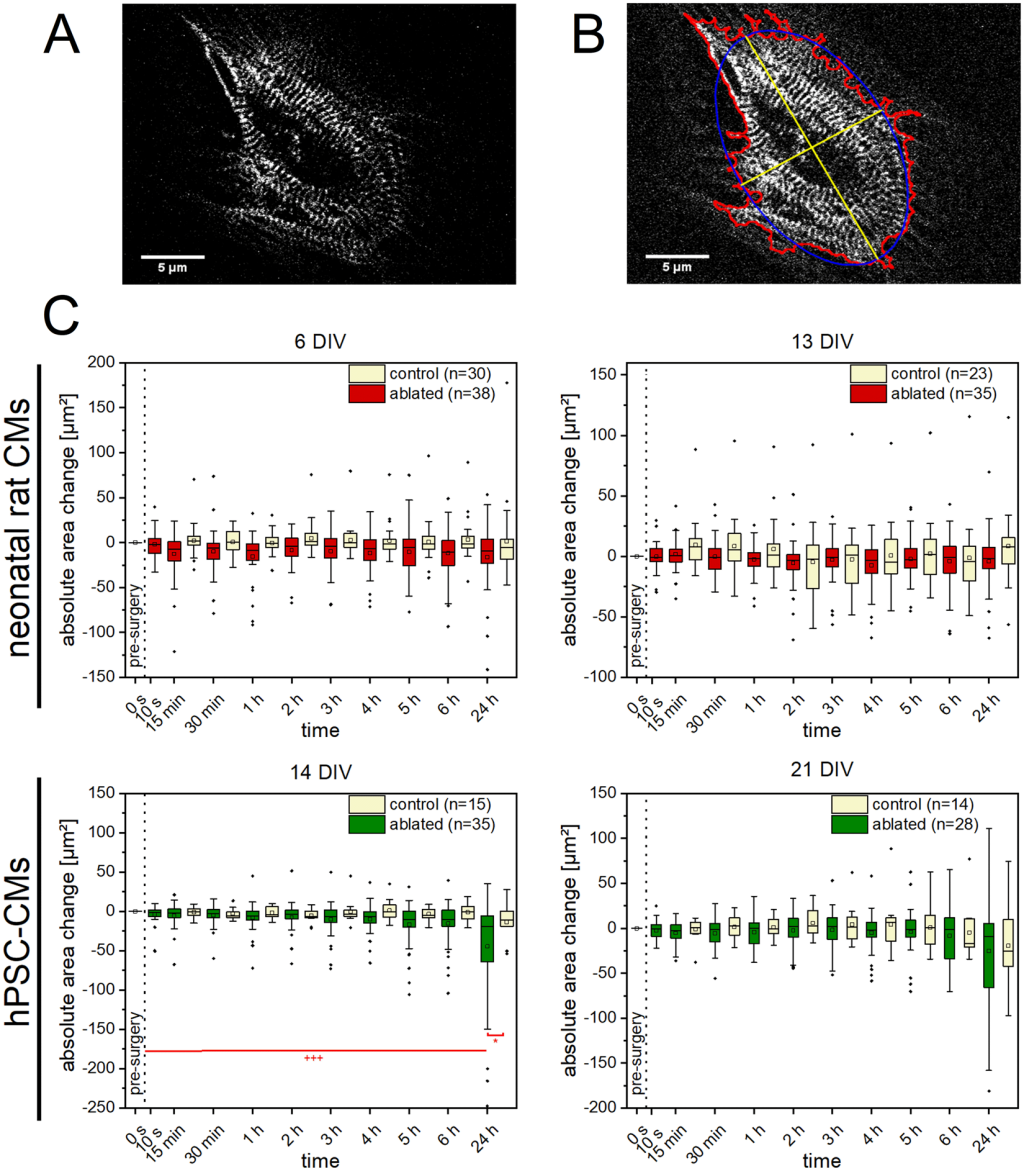
Morphological changes in CMs after single Z-disc ablation. Multiphoton images of turboRFP linked α-actinin expressing CMs. Here an hPSC-CM (**A**), was recorded before and frequently after single Z-disc ablation for a time period of 24 h. The cell area, perimeter (red) x- and y-expansion (yellow) were determined using a self-written ImageJ macro (**B**). Untreated CMs served as a control group. A significant decrease in cell area was observed for 14 DIV old hPSC-CMs (**C**). Significant differences between treated and control group (*) and between cell area of earlier time points (+) were found. Upper line of box, 75th percentile; lower line of box, 25th percentile; horizontal bar within box, median; upper bar outside box, 90th percentile; lower bar outside box, 10th percentile. Dots represent outliers. *P < 0.05, +++ P < 0.001.

We observed no significant changes of the cell area within a time period of 24 h for treated neonatal rat CMs (Fig. 4). However, 14 DIV old hPSC-CMs exhibited a significant decrease of cell area after 24 h compared to the control group as well as to the initial cell area before fs laser treatment. This was observed for all time points in 14 DIV old hPSC-CMs. In addition, a significant decrease of cell’s x- and y-expansion was found for the 24 h point in time in 14 DIV old hPSC-CMs (Supplementary Figs S3 and S4). Moreover, 6 DIV old neonatal rat CMs showed a significant decreased x-expansion for the 24 h point in time compared to the control group.

The cell perimeter showed no significant changes, neither in neonatal rat CMs nor in hPSC-CMs (Supplementary Fig. S5). Only small fluctuations around the initial values and comparable to the untreated control CMs were observed.

### Calcium oscillations are not elevated after Z-disc loss

Calcium is essential for myocyte contraction via Ca^2+^ dependent induction of conformational changes in the troponin-tropomyosin complex and is also a critical secondary messenger in major cellular pathways, including stress signaling^36^. We investigated calcium homeostasis of the CMs because single Z-disc loss potentially leads to cell stress and could affect cell contraction.

**Figure 5:**
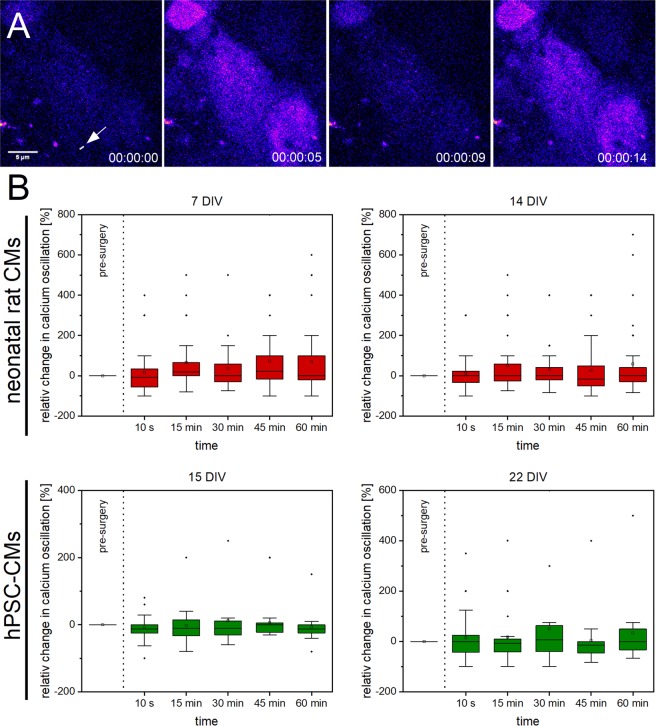
Calcium oscillations in CMs after single Z-disc ablation. Representative multiphoton time stack of an hPSC-CM stained with Fluo 4-AM (**A**, pseudocolor). The intracellular calcium level correlates with the Fluo 4 fluorescence intensity from which the calcium oscillations before and after single Z-disc ablation were determined. The relative changes in calcium oscillations over time are visualized in box plot graphs (**B**). Calcium oscillations in CMs before single Z-disc ablation served as reference value. Upper line of box, 75th percentile; lower line of box, 25th percentile; horizontal bar within box, median; upper bar outside box, 90th percentile; lower bar outside box, 10th percentile. Dots represent outliers. 7 DIV n = 33, 14 DIV n = 32, 15 DIV n = 24, 22 DIV n = 21.

We stained CMs with Fluo 4-AM to visualize calcium oscillations and recorded the Fluo 4 fluorescence intensity before and after single Z-disc ablation over time (Fig. 5). Using a self-written Matlab program, we observed a small increase in calcium oscillations in 7 DIV old neonatal rat CMs with a maximum (median) of 22,5% after 45 min. For hPSC-CMs, a maximum (median) of 6,25% in 22 DIV old CMs after 30 min was found. No elevated calcium oscillations were detected in 14 DIV old neonatal rat and 15 DIV old hPSC-CMs. We also analyzed neighboring untreated CMs (Supplementary Fig. S6). In neighboring neonatal rat CMs, a slight increase in calcium oscillations for 7 DIV old CMs with a maximum (median) of 28% and a significant increase to 66% after 60 min in 14 DIV old CMs was detected. No increased calcium oscillations were found in neighboring hPSC-CMs.

## Discussion

Cardiomyocytes are specialized to rapidly generate force and movement via the turnover of chemical energy to mechanical energy. The frequent contractility is based on the Ca^2+^ dependent interaction of actin and myosin filaments, which are arranged in sarcomeres. The connecting interface of adjacent sarcomeres is the heterogenic Z-disc complex. The Z-disc is a nodal point for signaling pathways, the mechanosensation and force transduction in CMs. Over the last decades, many disease causing mutations in Z-disc related proteins were found^8,37^. We applied, for the first time, fs laser-based nanosurgery to physically ablate a distinct Z-disc in single CMs to mimic structural Z-disc damage. The fs laser as intracellular nanoscissors can operate without producing generalized cell injury, necrosis^38,39^ and decreased cell viability^40^.

As a single Z-disc is an essential module of the complex cytoskeleton in CMs, we expected cell metabolism and viability to be influenced after ablation. However, our measurements revealed that the destruction of a single Z-disc does not influence the cell viability. A single Z-disc loss could be compensated equally in neonatal rat and hPSC-CMs, independent of their maturation state. As CMs are terminal differentiated with a limited cell division capacity, a high stress resistance is crucial for long-term survival and physiological functions. Several studies revealed that high oxidative stress^41^, intrinsic calcium overload^42^ or mechanical stress^43^ can lead to apoptosis induction. The loss of a single Z-disc is not sufficient to induce these stress levels.

As the viability was nearly unaffected, we were interested if treated CMs could recover the loss of a single Z-disc. An effective endogenous repair of microdamage in the sarcomeric cytoskeleton is of vital importance for the lasting efficiency of the heart. Intense stretching during muscle contraction can result in micro injuries^44^. In contrast to the extensive regeneration ability of skeletal myocytes, the *de novo* regeneration capacity of the adult human heart, and probably also CMs, is limited^45^. Studies of mammalian hearts revealed a transient regeneration capacity which terminates postnatal after 7–12 days in rats^46^. Consequently, we expected an overall reduced intrinsic regeneration capacity after Z-disc ablation and a negative correlation with the CMs maturation state. Our results disproved this expectation, since the majority of viable CMs of both origins recovered a full Z-disc pattern within 24 h. The endogenous recovery in hPSC-CMs was more prominent compared to neonatal rat CMs. However, both cell types where cultured in their specific, pre-established media which may affect the regenerative capacity.

The endogenous recovery of a regular Z-disc pattern could be explained by *de novo* biosynthesis of sarcomeric proteins. However, recent data by Leber *et al*. suggest that such a repair process is independent of protein biosynthesis^47^. An alternative explanation for the recovery of the Z-disc pattern could be the restructuring of the remaining original periodic sarcomeres. To avoid collapse of CM contractility, intact Z-discs of remaining, undamaged sarcomeres could be connected to stabilize damaged regions. For instance, FilaminC has shown to be present as an early linking protein to provide mechanical support^47^, whereas F-actin is subsequently involved in later repair processes^44^. Compared to bleached α-actinin in the Z-disc (FRAP kinetics t_1/2_~200 s), the endogenous recovery process to a regular Z-disc pattern is significantly longer (>1–2 h) whereby the majority of treated CMs recovered after 5 h (hPSC-CMs) or 24 h (neonatal rat CMs).

As endogenous repair processes were observed during continuous CM contraction, we were interested if a Z-disc loss is accompanied by changes of the cell morphology and cytoskeleton. Morphological alterations are connected to ventricular hypertrophy and heart failure^48,49^. Data from our observation did not favor a significant correlation between endogenous repair and morphology in neonatal rat CMs. However, a significant long-term decrease in cell area in 14 DIV old hPSC-CMs after 24 h was detected. Changes in myocyte length to width ratio, as an adaptive process to chronic pressure overload^48^, where not found in tested CMs.

In addition to morphological properties, efficient intracellular calcium homeostasis is necessary for continuous rhythmic contractions. The Z-disc is an integrative framework for calcium sensitive proteins and in close proximity to voltage dependent L-type calcium channels, T-tubules or ryanodine receptors^50–52^. However, the detailed role of a single Z-disc in the Ca^2+^ kinetics is unknown. We hypnotized that the destructed Z-disc leads to cell stress, and therefore to an increased intracellular Ca^2+^ concentration, which could result in arrhythmias^53,54^. Our findings did not confirm these expectations, potentially do to a compensatory effect of the connections between CMs via gap junctions^55^. However, the data from untreated neighboring CMs also showed no significant changes, except for the 60 min point in time in 14 DIV old neonatal rat CMs.

In conclusion, this study investigated the precise ablation of a cardiomyocytes Z-disc for the first time using fs laser-based nanosurgery. Most CMs survived and compensated the loss of a single Z-disc. Furthermore, nearly half of the myocytes repaired their Z-disc pattern within 24 h, retaining continuous contraction and Ca^2+^ handling. A fundamental understanding of the role of single elements in the cytoskeleton network is the key for *in vitro* disease model assessment. This is very important in clinical settings, as future studies involving Z-disc ablation can help to investigate cell regeneration e.g. genetic disease backgrounds and/or after pharmacological treatment or other external challenges such as infections and inflammation. Currently, cardiovascular conditions are often analyzed by increasing or weakening the strength of cell contraction with inotropic agents. These analysis usually measure sarcomere shortening and contraction after pharmacological treatment, for example in hiPSC-CMs^56^. However, it is also important to reveal the effect of a specific agent on cardiac repair and regeneration. Our approach of Z-disc ablation in combination with our recent studies on improved hPSC-CMs maturation *in vitro*^57,58^ provides an ideal model to investigate CMs recovery scenario(s) and strategies for their improvement on the single cell level. Consequently, fs laser-based nanosurgery of the Z-disc will help deciphering endogenous repair mechanism and ultimately reveal novel ideas for therapeutic treatments.

## Methods

### Generation of hPSC-derived cardiomyocytes

Human PSC-derived CMs were generated by directed differentiation according to recent protocols from Kempf *et al*.^34,59^. Briefly, hPSCs grown on Matrigel (corning) in mTeSR medium (Stem Cell Technologies) were harvested using 40 µL/cm² Accutase for 5 min at 37 °C and inoculated as single cells at 5 × 10^5^ cells/mL in mTeSR1 supplemented with 10 µM Y-27632 for 4 days in suspension culture to induce aggregate formation either in low attachment 6 well plates or Erlenmeyer flasks agitated on an orbital shaker (Celltron) at 70 revolutions per minute (rpm) and incubated at 37 °C and 5% CO_2_. To induce differentiation, medium was changed to RPMI1640 (with 2 mM glutamine) supplemented with B27 without insulin (Life Technologies, referred to as RB-). CHIR99021 (from G. Dräger, Leibniz University Hannover (LUH)) was added at 7.5 µM for 24 h. IWP2 (Tocris or LUH) was added at 5 µM after 24 h or 72 h and maintained for 48 h, respectively. Medium was refreshed every 2–3 days thereafter. Insulin was added from day 7 onwards (referred as RB+). Typically, hPSC-CMs content of 85–95% was achieved at day 10–14 of differentiation; cardiomyocyte content was monitored by flow cytometry specific to anti-sarcomere-specific pan-MyHC antibody (1:20, MF 20, Hybridoma Bank), anti-cardiac Troponin T (1:200, clone 13–11, Thermo Scientific), anti-sarcomeric α-actinin (1:800, EA53, Sigma-Aldrich) and respective anti-IgG isotype controls (DAKO), followed by an incubation with CyTM5-conjugated secondary antibody (1:200, Jackson ImmunoResearch). Data were acquired on an Accuri C6 flow cytometer (BD Biosciences) and analyzed using FlowJo software (Flowjo, LLC). After dissociation and seeding of hPSC-CMs, cells were cultivated for 14 resp. 21 days (DIV) in RB+ (21–30 days post induction of differentiation) until experiments were performed.

### Neonatal rat cardiomyocytes isolation

Primary rat cardiomyocytes were isolated from neonatal Spraque-Dawley rats (P2–P5) of both sexes according to a modified protocol from Maass and Buvoli^60^. In short: two to five day old neonatal puppies were decapitated, disinfected and the hearts were isolated and minced in small pieces. The cardiomyocytes were isolated using a Trypsin/DNase digesting solution at room temperature. Cells were pelleted by centrifugation, resuspended in MEM Eagle medium (PAN-Biotech GmbH, Germany; supplemented with 5% fetal bovine serum, 100 U/mL penicillin/streptomycin, 0.1 mM bromodeoxyuridine and 1.5 µM vitamin B12) and filtered through a 40 µm cell strainer. After a preplating incubation for 90 min at 37 °C and 5% CO_2_ atmosphere, one million cardiomyocytes were seeded into 35 mm glass bottom dishes (Ibidi, Germany), which were coated with 0.1% gelatin. The next day, all dishes were washed thoroughly with DPBS. The experiments were in accordance with the German Animal Welfare Legislation (§4, TierSchG) and approved by local authorities (Zentrales Tierlaboratorium, Laboratory Science, Hannover Medical School) and reported regularly. The heart isolation was permitted by the Lower Saxony State Office for Consumer Protection and Food Safety (reference number 42500/1 H).

### Cell culture and transduction

hPSC-CMs were cultured in RB+ medium (RPMI1640 with B27 supplement including insulin; Life Technologies, USA) and 100 U/mL penicillin/streptomycin. Rat CMs were cultured in MEM Eagle medium supplemented with 5% fetal bovine serum, 100 U/mL penicillin/streptomycin, 0.1 mM bromodeoxyuridine and 1.5 µM vitamin B12. The media where changed every three days. For imaging and manipulation, CMs were seeded in 35 mm glass bottom dishes (Ibidi, Germany), which were coated with 0.1% gelatin. Three days before nanosurgery, CMs were transduced with lentivirus particles (pLenti CMV Neo DEST alpha actinin RFP) to visualize Z-discs. pLenti CMV Neo Dest alpha actinin RFP was generated via Gateway® Cloning Technology (Invitrogen, USA) by cloning the expression construct tdTurboRFP-Alpha-Actinin-19 (Addgene plasmid #58050, Addgene, USA), gift from Michael Davidson, into the destination vector pLenti CMV Neo DEST (Addgene plasmid #17392), gift from Eric Campeau and Paul Kaufman. Viral particles were produced with a three-plasmid split packaging system as previously described^61^.

### Laser setup, imaging and manipulation

We used a Chameleon Ultra II laser system (Coherent Inc., Santa Clara, USA) with a pulse length of 140 fs and a repetition rate of 80 MHz for multimodal imaging and manipulation^32^. The laser power was attenuated using motorized half-wave plates (Thorlabs, Newton, USA) and polarizing beam splitter cubes (Thorlabs, Newton, USA). A LabView application (LLS ROWIAK) controlled two galvanometer-scanning mirrors and a shutter for precise multiphoton imaging and nanosurgery. The beam was focused via a 40x water immersion objective with a NA of 1.2. To maintain environmental parameters, the cell culture dish was placed on a 37 °C heated stage with CO_2_ perfusion. Imaging of turboRFP linked α-actin expressing CMs was performed at an excitation wavelength of 950 nm (pulse energy 0.25 nJ); Calcein and Fluo 4 imaging was performed at 850 nm (pulse energy 0.27 nJ). The turboRFP signal was detected with a photomultiplier tube and with an emission filter at 607 ± 18 nm, the fluorescence of Calcein and Fluo 4 was collected at 510–560 nm. For nanosurgery, a wavelength of 730 nm, a scanning velocity of 100 µm/s at 0.9 nJ over the chosen length was applied, revealed by previous experiments (Supplementary Fig. S7). All imaging data were analyzed using Fiji^62^.

### Z-disc ablation and morphology

TurboRFP-α-actinin expressing CMs were randomly selected and positions were saved. In a random portion of these CMs, a single Z-disc per cell was ablated, controlled by eye, and multiphoton images were recorded before and at 10 s, 15 min, 30 min, 1 h, 2 h, 3 h, 4 h, 5 h, 6 h, and 24 h after nanosurgery. The other randomly selected portion of untreated CMs served as a control group. By the help of a self-written ImageJ macro, the cell perimeter, area and x- and y-expansion was determined for the different time points and compared with the initial parameters. Briefly, the macro provided automated thresholding, identification of the cell borders, and morphological analysis (also see Fig. 4B).

### Determination of CM viability

TurboRFP-α-actinin expressing CMs were randomly selected and positions were saved. In some of the selected CMs, a single Z-disc per cell was ablated. Untreated CMs served as control. 24 h after single Z-disc ablation, CMs were stained with 2 µM Cacein-AM in culture medium for 20 min at 37 °C and 5% CO_2_ atmosphere to visualize cell metabolism. Nonspecific esterases, which are only present in viable cells, cleave the acetoxymethylester (AM) group of nonfluorescent Calcein-AM yielding the fluorescent and cell retained Calcein. Nonfluorescent cells were counted as dead cells. Cells with significant membrane protrusions were also counted as dead cells.

### Recovery of Z-disc pattern

For the determination of CMs regeneration potential the previous described time series of recorded images was analyzed. The recovery of a regular Z-disc pattern was determined by eye blindly by two independent researchers.

### Calcium homeostasis

Before Z-disc ablation, turboRFP-α-actinin expressing CMs were incubated with 2 µM of the calcium indicator Fluo 4-AM at 37 °C and 5% CO_2_ atmosphere. After 40 min, the culture medium was replaced and cells were incubated for 20 min at 37 °C and 5% CO_2_ atmosphere. Afterwards, CMs were randomly selected and positions were saved. The Fluo 4 fluorescence intensity was recorded (30 images with a frame rate of 2 Hz) before and 10 s, 15 min, 30 min, 45 min and 60 min after single Z-disc ablation. The fluorescence intensity of a selected region of interest near the ablation site was determined for all time points and standardized to the mean fluorescence intensity of the first image. After plotting of the standardized fluorescence intensity against the time, the number of calcium oscillation peaks was determined using a self-written Matlab (Matlab R2018a, The MathWorks) program.

### Data analysis and statistics

All data were graphically represented using the software Origin (OriginPro 2018b, OriginLab). Data were expressed as mean ± standard deviation (SD), and analyzed with a one-way ANOVA with repeated measures followed by Tukey,s test. P values of < 0.05 were considered as significant. Box plots characterized by upper line of box, 75th percentile; lower line of box, 25th percentile; horizontal bar within box, median; upper bar outside box, 90th percentile; lower bar outside box, 10th percentile. Dots represent outliers. All cells per dish were randomly selected and at least 4 independent dishes per experimental condition were used.

## Supplementary information

Supplementary Information

## Data Availability

The datasets generated during this study are available from the corresponding authors on reasonable request.
