# Supplementary material for: Femtosecond laser-based nanosurgery reveals the endogenous regeneration of single Z-discs including physiological consequences for cardiomyocytes
Source: Sci Rep. 2019 Mar 6;9:3625. doi: 10.1038/s41598-019-40308-z (PMC6403391; doi:10.1038/s41598-019-40308-z)
Supplement: Supplementary file 1 — Supplementary Information [file 41598_2019_40308_MOESM1_ESM.pdf]

Supplementary Information to:

Femtosecond laser-based nanosurgery reveals the endogenous regeneration of single Z-discs including physiological consequences for cardiomyocytes

Dominik Müller[1,2,3*], Dorian Hagenah[1,2,3], Santoshi Biswanath[2,4], Michelle Coffee[2,4], Andreas Kampmann[3,5], Robert Zweigerdt[2,4], Alexander Heisterkamp[1,2,3], Stefan M. K. Kalies[1,2,3]

[1]*Institute of Quantum Optics, Leibniz University Hannover, Hannover, Germany*

[2]*REBIRTH-Cluster of Excellence, Hannover Medical School, Hannover, Germany*

[3]*Lower Saxony Centre for Biomedical Engineering, Implant Research and Development (NIFE), Hannover, Germany*

[4]*Leibniz Research Laboratories for Biotechnology and Artificial Organs (LEBAO), Department of Cardiac, Thoracic, Transplantation and Vascular Surgery (HTTG), Hannover Medical School, Hannover, Germany*

[5]*Clinic for Cranio-Maxillo-Facial Surgery, Hannover Medical School, Hannover, Germany*

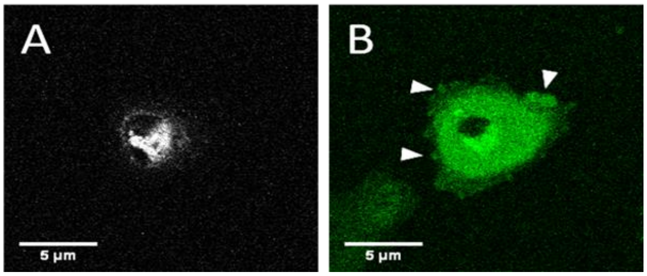

Supplementary Figure S1. Viability staining of a CM after single Z-disc ablation. In this representative image, an turboRFP linked α-actinin expressing hPSC-CM is shown 24 h after laser treatment (A). The metabolic activity was visualized using Calcein-AM staining (B) and a spherical morphology with blebs (arrow heads) on the cell membrane was observed. Scale bar 5 µm.

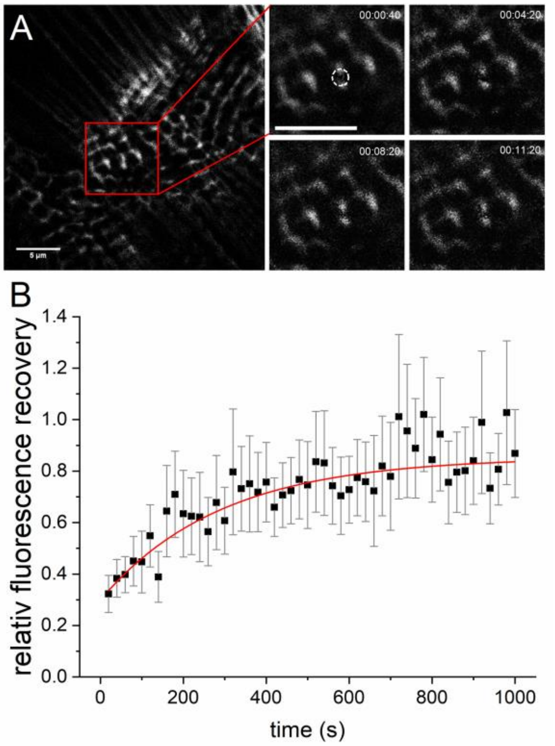

Supplementary Figure S2. Fluorescence recovery after photobleaching of a Z-disc. FRAP was performed in neonatal rat CMs after bleaching of turboRFP linked α-actinin in a single Z-disc. An exposure time of 5 ms at 100 % laser intensity on a Leica TCS SP5 confocal microscope was used. Cells were imaged afterwards using an excitation wavelength of 543 nm until the intensity of the bleached area saturated. (A) Image series representing the Z-disc pattern of an turboRFP linked α-actinin expressing neonatal rat CM before bleaching and at four time points post bleaching. A region of interest (dotted circle) was bleached. Post-bleach images were recorded at a time interval of 20 s. Scale bar 5 µm. (B) The fluorescence in the bleached region of interest was measured and normalized to the fluorescence intensity of an untreated Z-disc in the cell, including background subtraction. The intensity I(t) at every time point was averaged over nine measurements and is depicted with SEM. A single exponential growth $I(t) = I_0 + I_1 \cdot (1 - e^{-kt})$ with a bleaching

constant *k,* intensity offset I_0, and saturated intensity I_1 was used to fit the data (red line). The value of k indicated a half-recovery time of $\tau_{1/2} = \frac{\ln(2)}{k} = 200$ s.

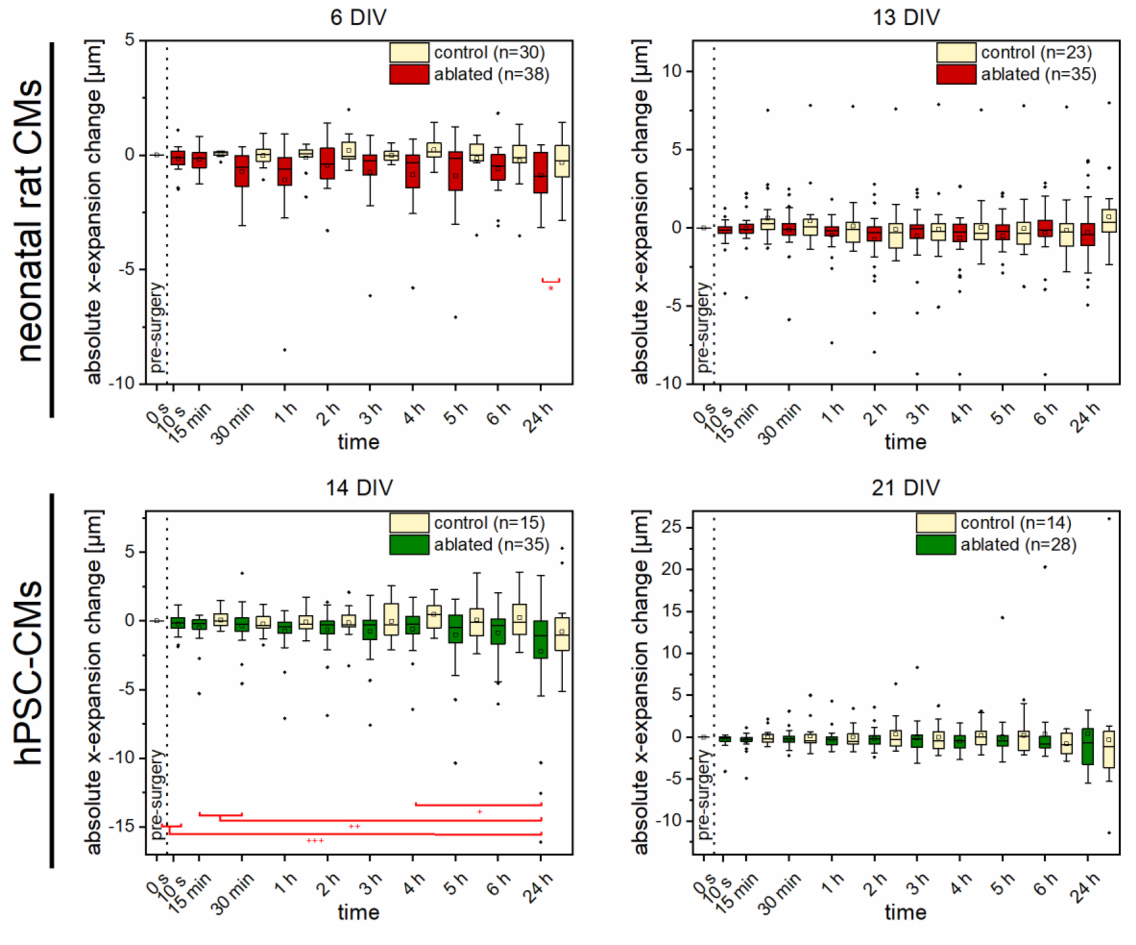

Supplementary Figure S3. X-expansion changes in CMs after single Z-disc ablation. Multiphoton images of CMs were recorded before and frequently after single Z-disc ablation for a time period of 24 h. The x-expansion was determined using a self-written ImageJ macro. Untreated CMs served as a control group. A significant decrease in cell's x-expansion was observed for 6 DIV old neonatal rat CMs compared to the control (*) and for 14 DIV old hPSC-CMs compared to earlier points in time (+). Upper line of box, 75th percentile; lower line of box, 25th percentile; horizontal bar within box, median; upper bar outside box, 90th percentile; lower bar outside box, 10th percentile. Dots represent outliers. *P<0.05, +P<0.05, ++P<0.01, +++P<0.001.

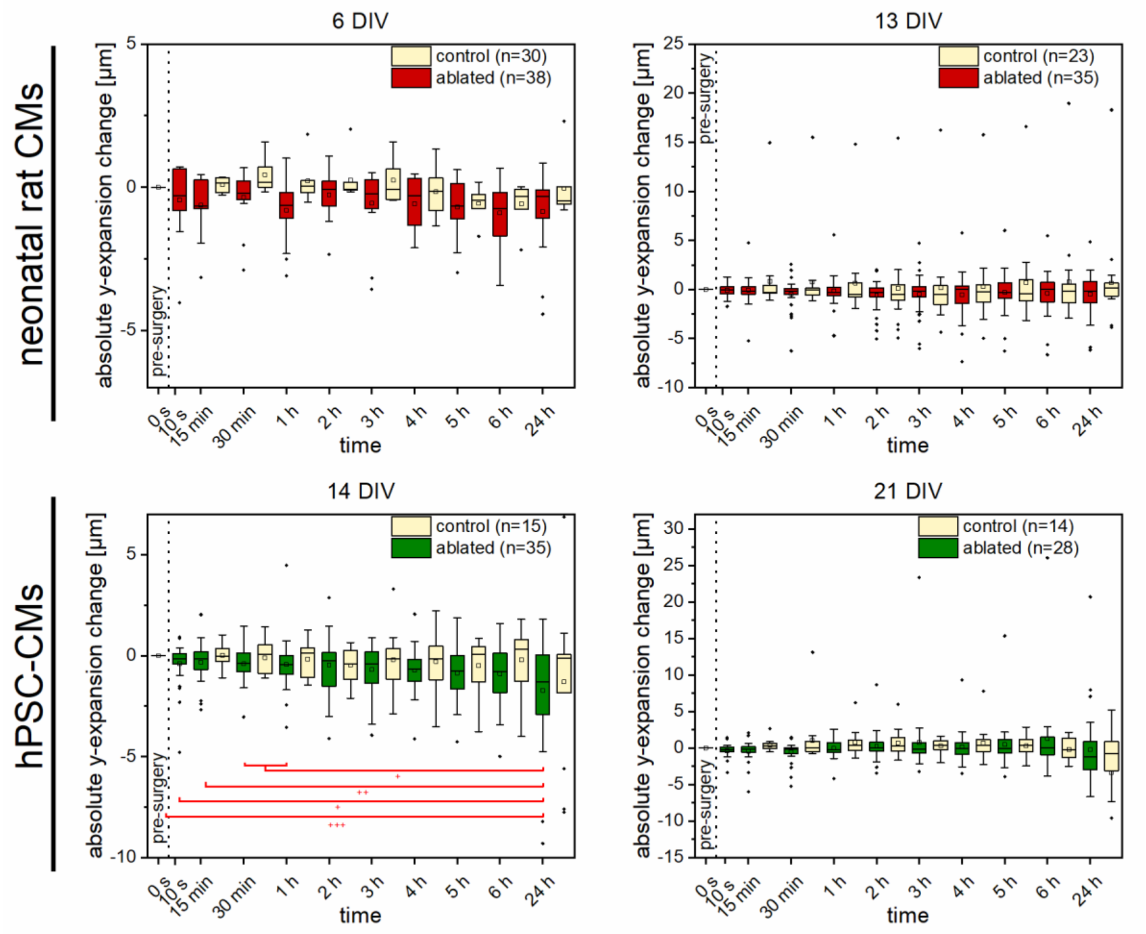

Supplementary Figure S4. Y-expansion changes in CMs after single Z-disc ablation. Multiphoton images of CMs were recorded before and frequently after single Z-disc ablation for a time period of 24 h. The y-expansion was determined using a self-written ImageJ macro. Untreated CMs served as a control group. A significant decrease in cell's y-expansion was observed for 14 DIV old hPSC-CMs compared to earlier points in time. Upper line of box, 75th percentile; lower line of box, 25th percentile; horizontal bar within box, median; upper bar outside box, 90th percentile; lower bar outside box, 10th percentile. Dots represent outliers. +P<0.05, ++P<0.01, +++P<0.001.

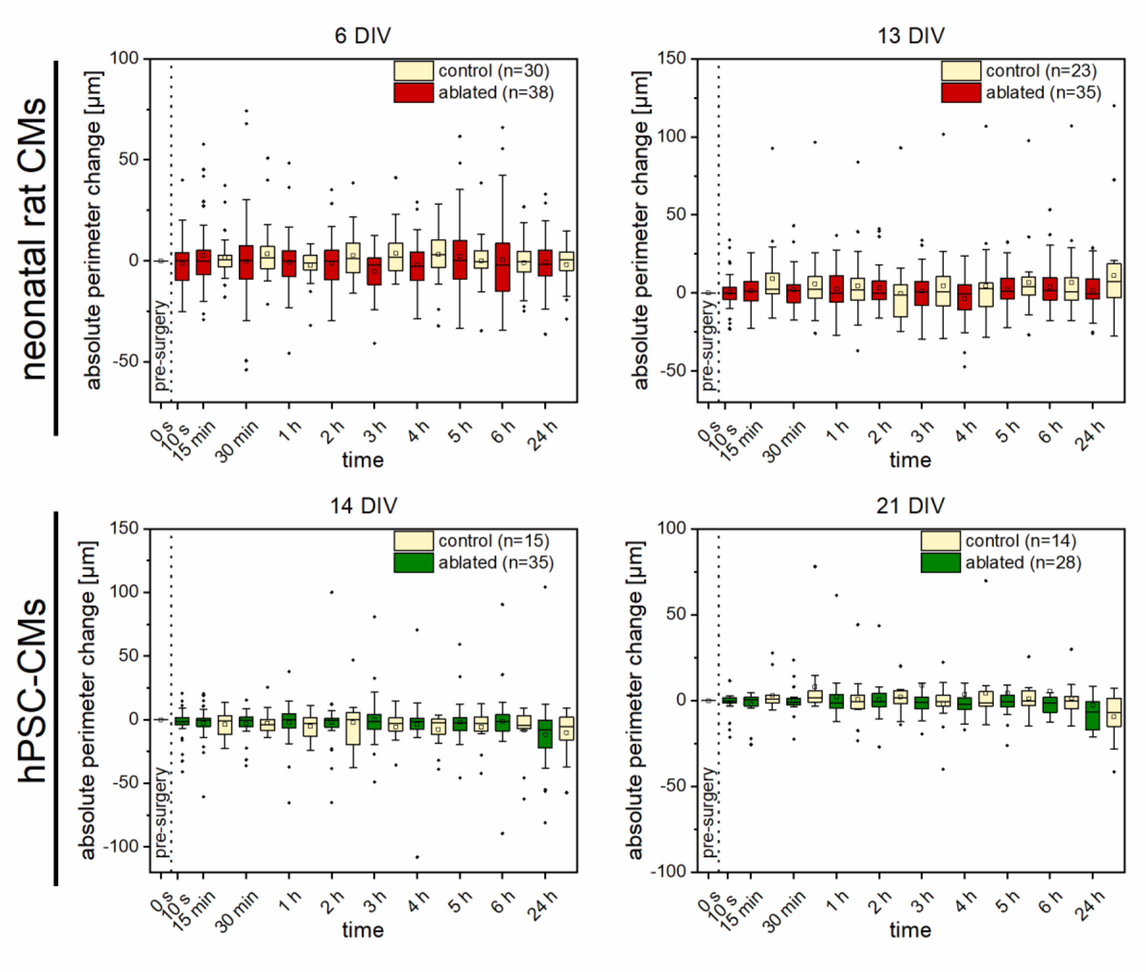

Supplementary Figure S5. Perimeter changes in CMs after single Z-disc ablation. Multiphoton images of CMs were recorded before and frequently after single Z-disc ablation for a time period of 24 h. The perimeter was determined using a self-written ImageJ macro. Untreated CMs served as a control group. Non-significant fluctuations in cell perimeter were observed for neonatal rat and hPSC-CMs. Upper line of box, 75th percentile; lower line of box, 25th percentile; horizontal bar within box, median; upper bar outside box, 90th percentile; lower bar outside box, 10th percentile. Dots represent outliers.

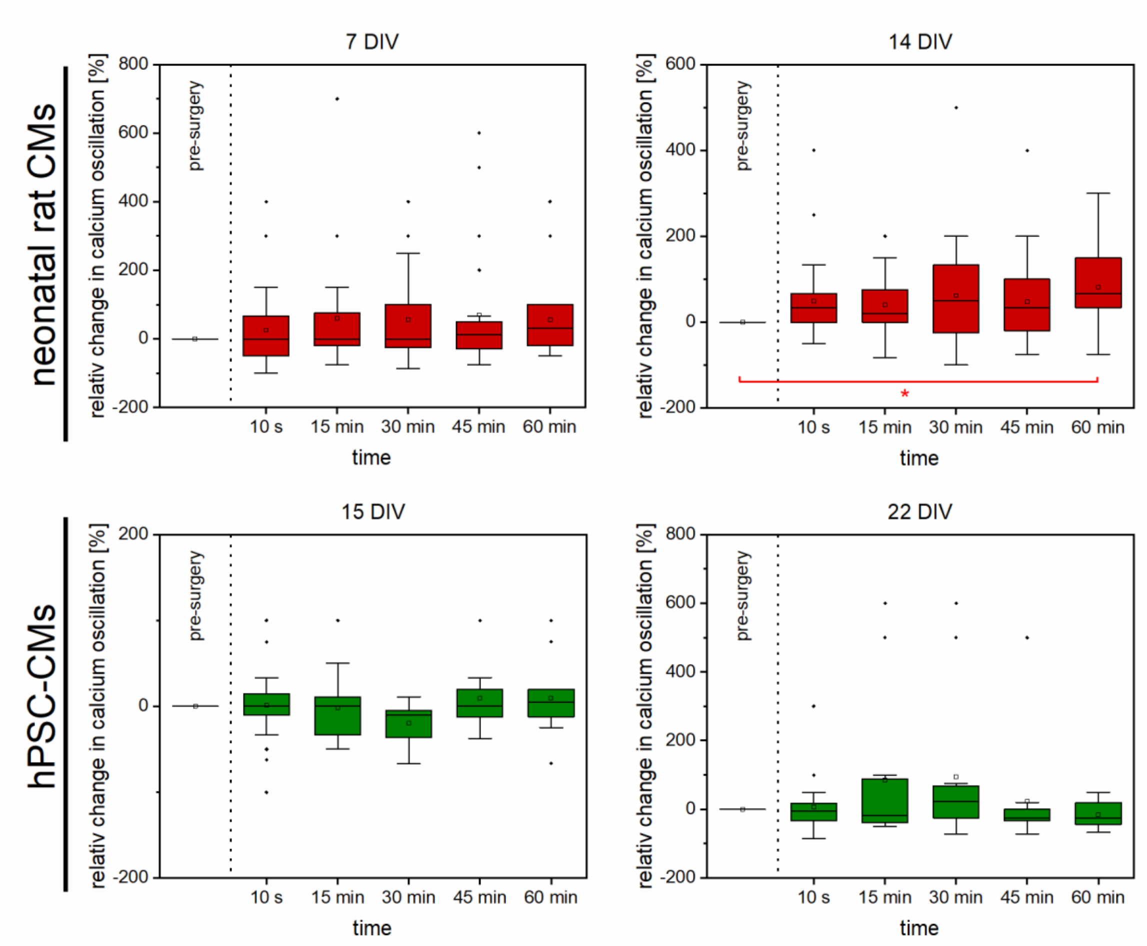

Supplementary Figure S6. Calcium oscillations in CMs after single Z-disc ablation in neighboring CMs. For the determination of calcium oscillations in CMs, adjacent to treated CMs, the previous described time series of recorded Fluo 4 images were analyzed. The fluorescence intensity of a selected region of interest in a CM, adjacent to the treated CM, was determined and plotted as described before. The relative changes in calcium oscillations over time are visualized in box plot graphs. Calcium oscillations in CMs before single Z-disc ablation in the neighboring CMs served as reference value. A significant increase in cell's calcium oscillations were found for 14 DIV old neonatal rat CMs compared to the control (*). Upper line of box, 75th percentile; lower line of box, 25th percentile; horizontal bar within box, median; upper bar outside box, 90th percentile; lower bar outside box, 10th percentile. Dots represent outliers. 7 DIV n=27, 14 DIV n=25, 15 DIV n=20, 22 DIV n=17. *P<0.05.

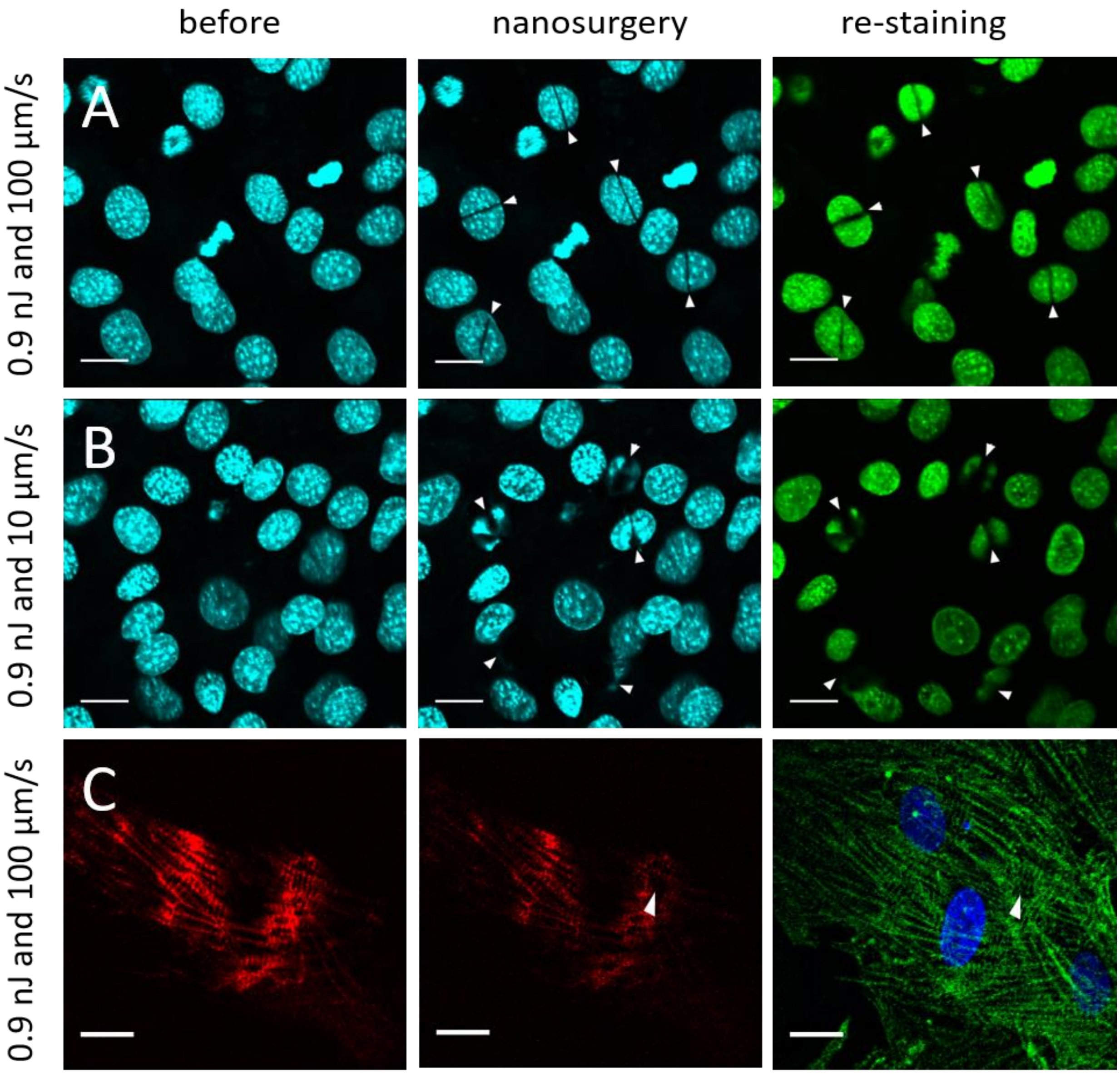

Supplementary Figure S7. Validation of laser parameters for nanosurgery. To determine the optimal ablation parameters, undifferentiated C2C12 cell nuclei were visualized with Hoechst (cyan, 2 µg/mL), dissected with a wavelength of 730 nm and re-stained with SYBR-Green (green, 2 µg/mL). For ablation, a laser power of 0.9 nJ and a scanning velocity of 100 µm/s (A) resp. 10 µm/s (B) were applied (indicated by arrowheads). As scanning velocities of 10 µm/s led to undefined nuclei damage, a scanning velocity of 100 µm/s was applied for further experiments. Images in C show a representative recording of a turboRFP linked α-actinin expressing neonatal rat CM before and directly after the Z-disc ablation (arrowhead) with a scanning velocity of 100 µm/s. The treated CM was fixed with 4 % PFA and re-stained via immunofluorescence labeling with anti-α-actinin (1:250, EA-53, Sigma-Aldrich) as primary- and Alexa Fluor 488 as secondary antibody (green, 5 µg/mL, A-10680, Invitrogen). Cell nuclei (blue) were visualized with Hoechst (2 µg/mL). A clear destruction of the regular Z-disc pattern is visible. Scale bar 5 µm.
